# Time-Resolved Photoluminescence Spectroscopy and Imaging: New Approaches to the Analysis of Cultural Heritage and Its Degradation

**DOI:** 10.3390/s140406338

**Published:** 2014-04-02

**Authors:** Austin Nevin, Anna Cesaratto, Sara Bellei, Cosimo D'Andrea, Lucia Toniolo, Gianluca Valentini, Daniela Comelli

**Affiliations:** 1 Istituto di Fotonica e Nanotecnologie—Consiglio Nazionale delle Ricerche (IFN-CNR), Dipartimento di Fisica, Politecnico di Milano, Piazza Leonardo da Vinci 32, 20133 Milano, Italy; E-Mail: sara.bellei@polimi.it; 2 Dipartimento di Fisica, Politecnico di Milano, Piazza Leonardo da Vinci 32, 20133 Milano, Italy; E-Mails: anna.cesaratto@polimi.it (A.C.); cosimo.dandrea@polimi.it (C.A.); gianluca.valentini@polimi.it (G.V.); daniela.comelli@polimi.it (D.C.); 3 Center for Nano Science and Technology@PoliMi, Istituto Italiano di Tecnologia, Via Giovanni Pascoli 70/3, I-20133 Milano, Italy; 4 Dipartimento di Chimica, Materiali ed Ingegneria Chimica “G. Natta”, Politecnico di Milano, via Mancinelli 7, 20131 Milano, Italy; E-Mail: lucia.toniolo@polimi.it

**Keywords:** fluorescence lifetime imaging (FLIM), time-resolved fluorescence spectroscopy, cultural heritage, degradation, monitoring, semi-conductor pigments

## Abstract

Applications of time-resolved photoluminescence spectroscopy (TRPL) and fluorescence lifetime imaging (FLIM) to the analysis of cultural heritage are presented. Examples range from historic wall paintings and stone sculptures to 20th century iconic design objects. A detailed description of the instrumentation developed and employed for analysis in the laboratory or *in situ* is given. Both instruments rely on a pulsed laser source coupled to a gated detection system, but differ in the type of information they provide. Applications of FLIM to the analysis of model samples and for the in-situ monitoring of works of art range from the analysis of organic materials and pigments in wall paintings, the detection of trace organic substances on stone sculptures, to the mapping of luminescence in late 19th century paintings. TRPL and FLIM are employed as sensors for the detection of the degradation of design objects made in plastic. Applications and avenues for future research are suggested.

## 1. Introduction

This work presents a review of the analysis of cultural heritage using time-resolved photoluminescence spectroscopy (TRPL) and fluorescence lifetime imaging (FLIM) which we illustrate through applied case studies. Both techniques are non-destructive and based on the use of ps or ns pulsed lasers and gated detection for the analysis of range of organic and inorganic materials.

### 1.1. Background of Photoluminescence Analysis of Works of Art

The analysis of works of art often begins with the visual examination of the surface of an object under UV light. This is because the spectrum of the optical emission from the surface as well as its spatial distribution in a field of view can provide conservators, art historians, and scientists key information regarding the presence of heterogeneities on a painting or a sculpture, signed papers or modern design objects. While the interpretation of fluorescence and the attribution of emissions to specific materials is far from trivial, both the spectrum of the emission, perceived as colour, and the spatial distribution of fluorescence are valuable starting points for further investigations. For example, conservators are experienced at relating differences in the fluorescence of surfaces to damage, to traces of materials (for example organic binders) which may provide insights regarding degradation or to past interventions, to the local applications of varnish (which tends to develop fluorescence with age) or to the presence of retouching (which is often dark when examined under UV light). Many materials found in cultural heritage fluoresce: indeed, stone substrates, organic pigments, binding media and waxes, conservation materials and semiconductors pigments have all been studied using fluorescence spectroscopy [1–10].

The luminescence from cultural heritage has long been utilized during routine inspection of paintings because it can be excited easily, with simple and low cost devices (lamps or LEDs); it is non-invasive, and can allow a reliable assessment of condition and the selection of suitable sampling locations for point-like analyses or sampling. The visual examination of works of art relies on the careful choice of both filtered UV-illumination and high-sensitivity color camera, providing a method for conservators to detect materials which may not be visible under normal lighting conditions. Typically, proper UV excitation is obtained with low-pressure UV lamps shielded with UV filters for suppressing the visible emission from the lighting devices; in these conditions, digital and analogue photography can provide spectacular images, as has recently been demonstrated during the analysis of wall paintings by Giotto in the Peruzzi Chapel (in the Basilica of Santa Croce, Florence, Italy) [11], where traces of original organic materials employed for paint and for gilding were revealed for the first time.

Many applications in the examination of works of art require the analysis of more quantitative parameters of the emission, including the emission spectrum of a material which reflects its chemical composition. The modification of the fluorescence of organic materials has been reported and related to general and more specific molecular changes, including those related to oxidation phenomena: for example, the photooxidation of protein-based binders [12], oils and varnishes [2], or the oxidation of modern polymers and plastics [13]. It is recognized, however, that the discrimination of materials on the basis of fluorescence spectra is often impossible—subtle spectral differences, which may arise from chemical modifications of materials or differences in molecular properties, may be masked by competing effects, auto-absorption phenomena [14], or scattering [15], for example. Fluorescence emissions may also be weak and thus spectra may be difficult to detect.

### 1.2. Time-Resolved Photoluminescence

In addition to the emission spectrum recorded from the surface of an object, the dynamics of the fluorescence, or luminescence, emission can be useful in the analysis and monitoring of cultural heritage and cultural heritage materials, which is the focus of the analysis presented in this article.

In simple terms, the emission process consists of the radiative decay from excited states of the chromophore. The emission lifetime can be interpreted as the average time the fluorophore stays in the excited state and hence provides information on the emission dynamics [16]. According to the nature of the excited state (singlet or triplet state) the lifetime can be extremely different varying from ps to ms. In the first case we generally refer to the emission process as fluorescence, while in the second case as phosphorescence. Both phenomena are generally summarized under the term luminescence.

Decay channels, from excited to ground state, can be classified as radiative and non-radiative. The emission decay depends on both radiative and non-radiative processes, because both of them influence the excited state population, leading to a strong dependence of the lifetime on the microenvironment of the fluorophore. In other terms, any interaction of fluorochromes with the microenvironment provides a specific emission quenching that is reflected by the emission lifetime. Therefore, this parameter carries clues about chemical changes (e.g., bond breaking) affecting the emitters due to oxidation, aging and other modifications of organic molecules. This represents one of the main advantages provided by the measurement of the fluorescence lifetime. Moreover, some emitters (e.g., fluorescent pigments) show specific fluorescence lifetimes, which can be considered characteristic.

Intensity measurements, especially those which are spectrally resolved, are affected by several drawbacks: first the presence of absorbers can severely extinguish the fluorescence signal and distort recorded spectral features. This is mainly true when dealing with paintings, because colors in the painted layer can strongly modify the emission from organic binders, which are often the main subject of the scientific investigation. Further, intensity measurements are affected by the spatial distribution of the excitation light, which is typically uneven, and by ambient light, which can seldom be avoided, unless measurements are done in complete darkness in the laboratory. For these reasons, fluorescence images may often be misinterpreted because areas with comparable chemical features appear different because of artifacts. In contrast the relaxation dynamics of the fluorescence or phosphoresce emission, like the tone of a sound, are almost insensitive to the intensity of the signal, provided that they can be reliably measured. Finally, time-resolved measurements are, at least on the first order, insensitive to ambient light since any continuous wave light, being uncorrelated with excitation pulses, gives a negligible contribution to the signal in the very low duty cycle measurement gates, provided that the repetition rate used is below few kilohertz.

### 1.3. Overview of the Lifetime of Luminescent Materials in Cultural Heritage

Different organic materials can be found on cultural heritage objects, which include protein and oil-based binders, varnishes, restoration treatments, adhesives and glues. These materials are often luminescent due to the presence of delocalized electrons in molecules containing multiple aromatic rings or long-chains of conjugated double bonds. The related decay kinetics associated with these molecules is on the order of picoseconds or nanoseconds [1] and is highly affected by a number of factors, which include pH, temperature, solvent polarity and molecular flexibility. Similar lifetimes characterize the emission from natural organic pigments, such as lakes or diazo pigments, and synthetic organic dyes (including phtalocyanines and anthraquinones) [17,18].

A different decay behavior is detected in luminescent inorganic materials typically present on cultural heritage objects. For example, semiconductor pigments, such as cadmium- and zinc-based pigments, are typically characterized by a fast picosecond band gap emission, due to the recombination of an electron with a hole from the conduction to the valence bands. Moreover, trap state levels, present in these emitting molecules as a consequence of intrinsic and extrinsic defects, give rise to further radiative relaxation decay paths with a much longer temporal scale (typically on the order of microseconds) [19].

An example of luminescent materials employed in contemporary art is provided by “glow-in-the-dark” paints. These pigments are based on long-living phosphors, such as copper- or silver-activated zinc sulfide and, more recently, doped strontium aluminate, and typically glow a pale green to greenish blue color which can last for up to hours after exposure to UV excitation. A further interesting example of luminescent material is cuprorivaite, which forms the basis of the synthetic blue pigment first produced in the 4th Dynasty in Ancient Egypt, known as Egyptian blue. The pigment is strongly luminescent, with the emission ascribable to Cu^2+^ ions in the crystal matrix and characterized by a peak in the infrared (at 910 nm) and a reported luminescence decay time of 107 μs [20]. Similar emitting properties has been reported for other luminescent inorganic pigments, as those based on BaCuSi_4_O_10_ (Han blue) and BaCuSi_2_O_6_ (Han purple) [21].

The optical emission from stone sculptures and monuments is usually due to intrinsic defects in the mineral structure or to trapped impurities. For example in calcite, trace concentrations of ions from transition metals (Mn^2+^) and rare-earth elements (including Tm^3+^ and Eu^3+^) gives rise to phosphorescence emissions characterized by different decay kinetics (of the order of ms and μs for the former and the latter, respectively), which can be easily discriminated by time-resolved luminescence spectroscopy [10,22]. It has to be reported that, as marbles and stones are rather porous materials, the optical emission from stone artworks is also ascribed to adsorbed organic contaminants (including humic acids). Nevertheless, the different decay kinetics which characterize the intrinsic emission from stone and from exogenous organic contaminants, allow the easily discriminate between these two contributions, as will be better outlined in a case study discussed in a following section.

The values of typical emission lifetimes of luminescent materials are given in Table 1.

## 2. Methods

### 2.1. Fluorescence Spectroscopy and Fluorescence Imaging

Time-resolved fluorescence analysis requires pulsed excitation and gated detection. In this work femtosecond and nanosecond pulsed laser radiation at different wavelengths is employed for excitation. Detection for fluorescence lifetime imaging (FLIM) is provided by a time-gated imaging intensifier, while time-resolved photoluminescence spectroscopy (TRPL) relies on a picoseconds streak camera system.

Laser-induced fluorescence (LIF) has received significant attention for the analysis of pigments [3,6,23] and binding media [24,25]; indeed it has been employed for the measurement of the emission decay kinetics of samples only in few cases [8]. Laser excitation has distinct advantages over lamp illumination, such as brightness and monochromaticity, which allow fluorescence measurements without the need for filters to remove stray or parasitic blue radiation, as required when using mercury-based UV lamps. The choice of suitable excitation wavelength requires knowledge of the absorption properties of materials—for example, organic materials often absorb in the ultraviolet and emit in the visible range—while semiconductor pigments and trapped ions may be more efficiently excited with visible radiation and may emit in the visible and infrared region [15,26].

### 2.2. Description of Fluorescence Lifetime Imaging (FLIM)

The fluorescence lifetime imaging (FLIM) apparatus is comprised of a ns laser excitation source combined with a time-gated intensified camera (C9546-03, Hamamatsu Photonics, Hamamatsu City, Japan), capable of high speed gating to capture images of transient phenomena. A custom-built trigger unit and a precision delay generator (DG535 Stanford Research System, Sunnyvale, CA, USA) complete the system, giving rise to a net temporal jitter close to 0.5 ns.

The choice of the laser depends on the absorption properties of the investigated sample. Usually, a Q-switching frequency-tripled diode-pumped Nd:YAG laser (FTSS 355-50 Crylas GmbH, Berlin, Germany, λ = 355 nm, Pulse energy = 70 μJ, Pulse duration = 1.0 ns) is used in order to excite luminescence from materials absorbing in the UV range; different excitation wavelengths can be achieved by using a compact dye laser optically pumped by the UV laser radiation (FTSS Dye Lasers, Crylas GmbH, Berlin, Germany).

The laser beam is coupled to a silica optical fiber; the fiber tip is magnified with suitable optics in order to uniformly illuminate a circular area of about 25 cm in diameter, leading to a typical fluence per pulse kept below 140 nJ/cm^−2^ (well below any damage threshold).

**Figure 1.**
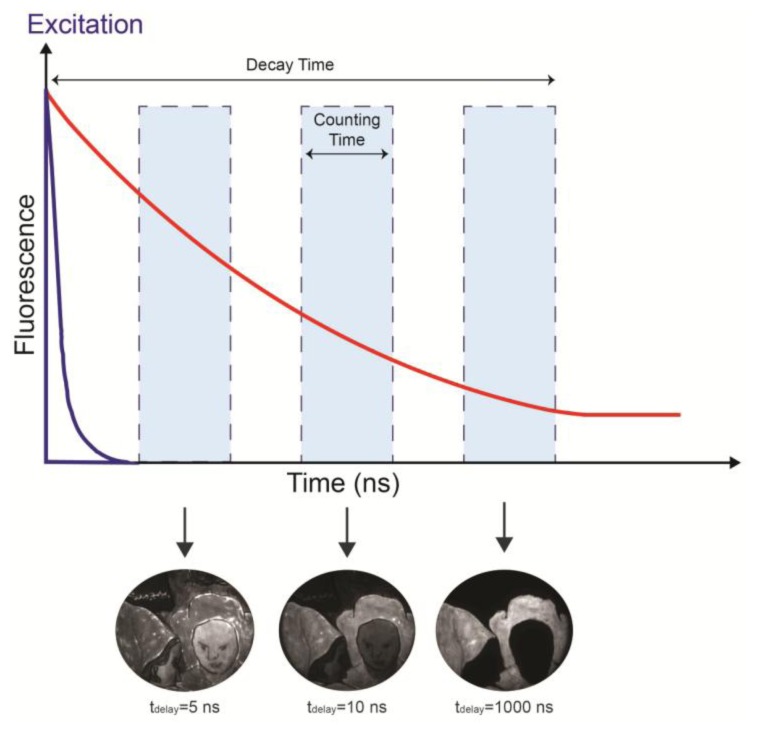
A timing diagram showing pulsed laser excitation, luminescent emission, and operation of the intensifier gate. The acquisition of luminescent images at different delays with respect to the laser pulse (purple). The intensity of the luminescence (red) decreases in time. The images recorded highlight the presence of a long-lived emission (>1,000 ns). Intensity in insets has been rescaled to fill the dynamic range of each image.

The fluorescence decay is temporally sampled by the image intensifier, whose gate width is adjustable from 3 ns to continuous mode, depending on the kinetic properties of the sample or surface under investigation. Usually, a 10 ns gate width is applied to detect the nanosecond kinetic of the emission from organic materials, but long-lived decay kinetics, on the order of the microsecond and millisecond, can be more effectively sampled by increasing the width of the gate window. The fluorescence signal within the gate window is then intensified by the MCP intensifier and accumulated by a CCD camera (QImaging Retiga 2000R fast, Cooled Mono 12-bit, Surrey, BC, Canada). This procedure is repeated at different delays between the excitation pulses and the leading edge of the gate, thus leading to a sequence of fluorescence images taken at different times (Figure 1).

The FLIM device is not spectrally resolved: in fact the image detector, based on an input multialkali photocathode, allows the detection of all photons emitted in the 400–900 nm spectral range. In order to select emission from specific spectral regions, a suitable band pass optical filter can be mounted in front of the image intensifier. The whole system has been assembled in a portable rack of about 60 × 60 × 70 cm, except for the gated camera that is connected to the control unit through a 10 m cable and a 40 m fiber optic, for remote access during in situ investigations. The long optical fiber allows an easy synchronization of the electronic gate with laser pulses in an almost jitter free configuration.

The lifetimes are subsequently calculated pixel-by-pixel, and then their spatial variations are displayed in a false colour representation. As a general feature, the more time samples are taken, the better is the estimate of the lifetime. Luminescence can seldom be modeled as a mono-exponential decay due to the simultaneous presence of several fluorescent species and different non-radiative relaxation paths. Nevertheless, the reconstruction of the effective lifetime map based on a simple mono-exponential decay model is typically the optimal choice for our investigations [27]. In fact, it leads to a single map that yields strong spatial contrast for the discrimination of different compounds and benefits from intensity independence. Moreover, the mono-exponential fitting algorithm is suitable for real-time data processing. Refined models (bi-exponential decay, multi-exponential decay, stretched exponentials) can be also applied to time-resolved data. Nonetheless, an extensive dataset and a much longer fitting time are required and still the fitting may be compromised by insufficient signal-to-noise ratio of data.

### 2.3. Time-Resolved Photoluminescence Spectroscopy

**Figure 2.**
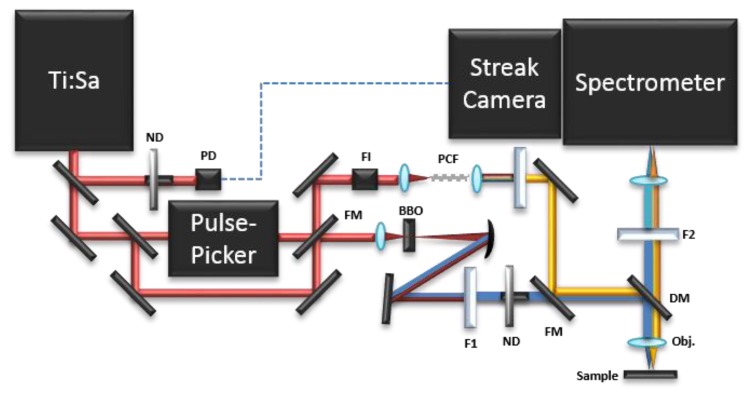
The TRPL setup. ND: neutral density attenuator; PD: photodiode; FI: Faraday isolator; PCF: photonic crystal fiber; FM flipping mirror; BBO: type I β-barium borate crystal; F1: low pass filter; DM: dichroic mirror; Obj.: microscope objective; F2: high pass filter.

Time-resolved photoluminescence (TRPL) analysis consists in the measurement of the evolution of the luminescence spectrum over time. In contrast to FLIM, TRPL spectroscopy studies the luminescence emission from a single point in the sample and not from an entire image. Different set-ups can be built to perform TRPL analysis. All of them combine: (a) a pulsed laser source; (b) a triggering system; (c) a proper optical path to deliver the laser pulse to the sample; (d) light collecting optics; (e) a spectral dispersive element; (f) a detector unit. In particular, in Conservation Science, a time-gated Optical Multichannel Analyzer (OMA) [27,28], a fast streak camera based system [28], and a portable Time-Correlated Single Photon Counting (TCSPC) apparatus [17] have been employed in the past for the analysis of the spectrally-resolved luminescence decay kinetics of pigments and binding media. The time-resolved photoluminescence (TRPL) measurements reported in this article were carried out using streak camera-based instrumentation. A scheme of the set-up is shown in Figure 2.

The pulsed excitation source consists of a passive mode-locking Ti:Sapphire laser (Chameleon Ultra II, Coherent, Santa Clara, CA, USA) tunable from 680 to 1,080 nm and emitting 140 fs pulses with a maximum energy of about 50 nJ. The laser repetition rate is 80 MHz and can be reduced down to 1 kHz by letting the beam pass through an acousto-optical modulating pulse picker (APE). To extend the excitation wavelength into the UV and visible ranges, a 1 mm type I β-barium borate crystal frequency-doubles the optical signal (340–540 nm). By using of a thin absorbing filter (BG38, Schott AG, Mainz, Germany) the residual fundamental is removed. In order to cover the remaining gap in the visible wavelength range (540–680 nm), a pulsed supercontinuum (SC) is generated by focusing the light pulses at 780 nm, by means of an aspheric singlet lens (C150TM, Thorlabs GmbH, Dachau, Germany), into a 20 cm long Photonic Crystal Fiber (PCF) (NL-2.4-800, Blaze Photonics Limited, Blaze Photonics Limited, Bath, UK). PCF consists of a silica fiber with a core (diameter of about 2.4 μm) surrounded by a mesh of air-filled holes. SC is generated over the spectral range 480–1100 nm through a combination of several nonlinear phenomena in the PCF [29]. The desired wavelength range is sliced from the broad SC spectrum using a suitable interference filter. In order to avoid back reflection into the Ti:Sapphire cavity, a Faraday Isolator is used before focusing into the PCF.

The light pulses are delivered into a custom-built epi-fluorescence microscope based on a dichroic mirror, chosen on the basis of the excitation wavelength, and a proper long working distance objective lens. The fluorescence radiation is collected by the same objective, filtered by a proper long pass filter to remove residual excitation light, and focused into the entrance slit of an imaging spectrometer (focal length 300 mm, f/3.9, 50 lines/mm grating, Acton SP2300, Princeton Instruments, Trenton, NJ, USA) with a spectral resolution of about 1 nm. The spectrometer is coupled to a streak camera detector (C5680, Hamamatsu Photonics, Hamamatsu, Japan), triggered by delivering a small portion of the laser beam to a photodiode. The streak camera detector can work in two different modalities, according to the required temporal range and related temporal resolution. The highest temporal resolution of about 2 ps can be reached in synchroscan operation mode working at 80 MHz repetition rate [30]. When it is necessary to study longer time decays, the slow sweep acquisition mode can be employed, with a maximum temporal resolution of about 50 ps and a highest repetition rate of 2 MHz [31].

## 3. Case Studies

In the following section, a short review of applied cases is given to highlight the effectiveness of the time-resolved approach to the analysis of luminescence. Without any claim for completeness, the examples range from model samples and Renaissance paintings to modern pigments and polymeric materials, sharing a similar approach that reveals many advantages.

### 3.1. FLIM of Model Samples and Organic Materials

The analysis of organic materials has long received attention using fluorescence spectroscopy, and has included time-resolved approaches [3,8,32]. The analysis of binding media—which contain proteins (egg white, animal glue and casein) and lipids (drying oils), mixed media (egg yolk which contains both proteins and lipids), as well as varnishes—has suggested that differences in media are appreciable in time-resolved spectra. Different amino acids or degradation products in proteins, and the formation of conjugated bonds and chromophores in oils and resins give rise to the observed fluorescence and to different fluorescence lifetimes.

**Figure 3.**
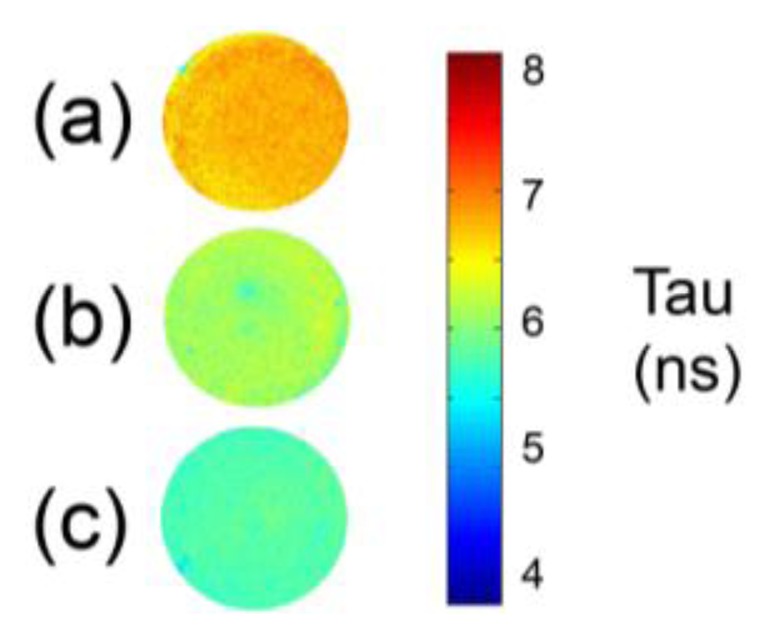
Fluorescence lifetime of (**a**) egg white; (**b**) collagen-based glue; (**c**) egg yolk.

The analysis of protein-based binding media like egg white, collagen-based glue and egg yolk, performed with FLIM can highlight differences in the fluorescence lifetime according to media. For example, while the fluorescence of egg white and collagen-based glues is greater than 5.5 ns, egg yolk, which also contains fatty acids, the oxidation of which gives rise to the formation of new fluorophores, is associated with a shorter fluorescence lifetime (Figure 3).

**Figure 4.**
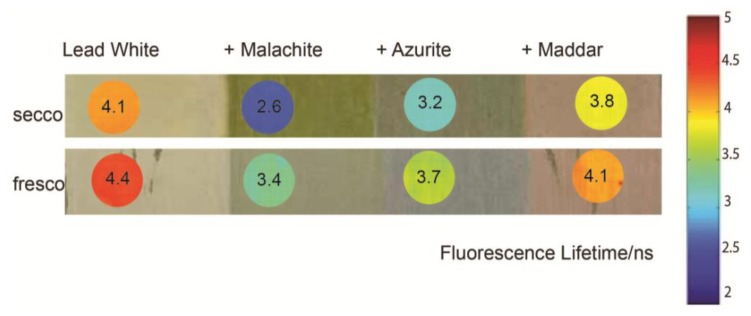
FLIM analysis (shown in false colour in circular insets) from a model sample painted in different mixtures of pigments in an egg yolk + linseed oil binder applied either to dry plaster (*secco*) or to fresh plaster (*fresco*).

A set of painted model samples containing whole egg mixed with linseed oil as the binder and lead white with significant concentrations of other pigments (copper-containing carbonate pigments and madder lake), painted *a fresco* and *a secco*, was analyzed with FLIM (Figure 4). The analysis of the model samples is of interest for a better comprehension of the interactions between a binder and the surrounding micro-environment. Indeed, the influence of pigments on the detected fluorescence depends on a number of factors which include the optical absorption of the pigment [15,33], possible luminescence of the pigment, and chemical interactions (e.g., quenching) between the binder and pigment and between the binder and the substrate [16]. Copper (II) ions are well-known quencher for fluorescence, and it has been shown that they influence the fluorescence spectrum of protein-based binding media [12]. Basic lead carbonate (2PbCO_3_·Pb(OH)_2_, lead white) can react with fatty acids to form lead soaps [34]. Madder lake is a fluorescent pigment which emits around 610 nm [35] and has a short fluorescence lifetime (on the order of few ns) [17,36].

Although the general shape of the spectra of the different samples containing egg-oil mixed with pigments is similar (data not shown), the fluorescence lifetime is different for each mixture. The presence of madder has the effect of reducing the fluorescence lifetime with respect to areas painted in lead white; this is ascribed to the contribution of emissions from the lake pigment, which, on its own has a lifetime of approximately 1.5 ns [17]. The copper-based pigments significantly reduce the fluorescence lifetime, and this could be due to the quenching of fluorescence by Cu^2+^ ions, which may occur by a Förster resonance energy transfer (FRET) mechanism [37]. The fluorescence lifetime of paint containing malachite is shorter than that observed in azurite-containing paint, which may be related to the slightly greater solubility of the pigment in the oil-egg mixture [38], as well as the greater concentration of malachite compared to azurite in the sample, resulting in an increased quenching of the binder emission lifetime. Finally, areas painted *a fresco* have increased fluorescence lifetime with respect to those painted *a secco*. The reason behind this phenomenon is not certain; however, it has been shown that calcium ions increase the fluorescence lifetime of amino acids and other fluorophores [39]. Therefore, it is possible that the increase in fluorescence lifetime in areas painted *a fresco* is due to the interaction between Ca^2+^ and fluorophores present in binding media.

### 3.2. Analysis of Pigments and Paintings

#### 3.2.1. Organic Lake Pigments

**Figure 5.**
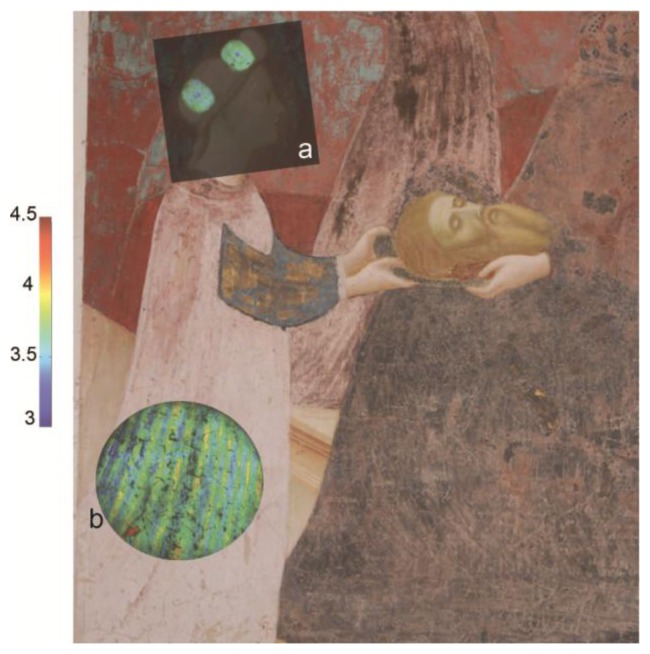
FLIM analysis of the wall painting of the Life of John the Baptist (Colleggiata, Castiglione Olona, Varese, Italy) by Masolino: the fluorescence lifetime maps taken from the figure of Salome were superimposed on the color image. In the ovals of the headdress (**a**) and in the vest (**b**) areas with different lifetimes can be discriminated, which are related to a different content of red lake.

The presence of an organic lake pigment was successfully mapped using FLIM on a Renaissance wall painting by Masolino da Panicale (1383–1447). The analysis was part of a three year monitoring campaign on the paintings of the Baptistery of Castiglione Olona (Varese, Italy) and it was combined with other advanced imaging techniques and with laboratory analyses on an historical micro-sample from the painted surface [40]. The FLIM maps were obtained from various areas, but the most interesting information came from the figure of Salome, as reported in Figure 5.

In particular, in Salome's headdress (shown in Figure 5a) it is possible to discern a dot in the center of two of the ovals. In these areas the lifetime is shorter (effective τ close to 3.1 ns) compared with the one detected in the surrounding area (effective τ close to 3.5 ns). The measurement of the lifetime is difficult to interpret and not strictly analytical by itself, but when combined with the spectrally resolved fluorescence imaging device and a photograph (taken in 1932 before the restoration conducted by Pellicioli and della Rotta) it allows us to understand more about the physical history of the wall paintings. The emission from the headdress was ascribed to an anthraquinone-based lake pigment mixed with an organic binder. The shorter emission lifetime from the dots was explained with a higher content of the red lake pigment with respect to the binder, added to the painting as *a secco* decorations, which are no longer visible to the naked eye. The hypothesis was extended to the dress worn by Salome (Figure 5b), which was probably decorated with same lake-based vertical stripes in order to give a tridimensional volume to the entire figure.

#### 3.2.2. Semi-Conductor Pigments Based on Cadmium and Zinc

During the 19th century, cadmium-based pigments were rapidly adopted by artists as they were bright and had a particularly high covering power [41]. Different shades of cadmium pigments were available and varied from light yellow to deep red on the basis of the chemical composition: the lighter colours are based on cadmium sulphide ((Cd,Zn)S), while the deeper shades are based on cadmium sulphoselenide (Cd(S,Se)). The two compounds belong to the IIb-VIa semiconductor group and are characterized by a large band gap energy and a direct transition [19,42].

**Figure 6.**
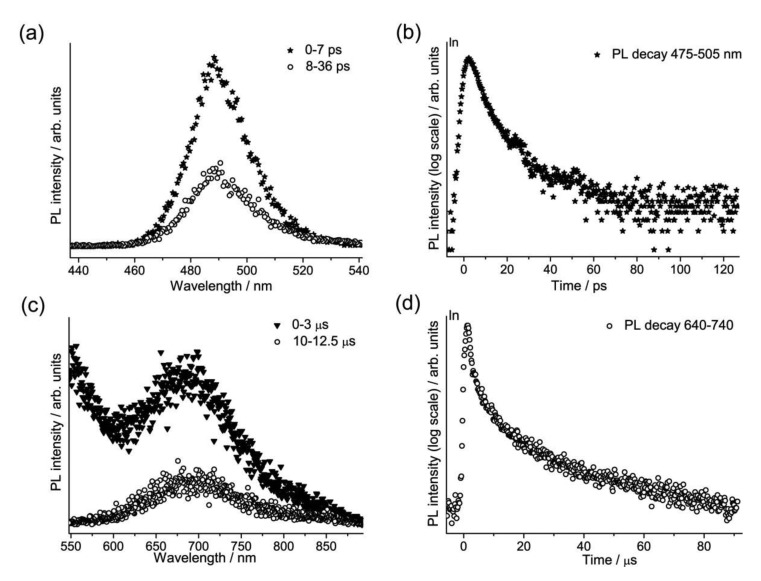
TRPL analysis of a yellow ((Zn,Cd)S) sample: gated PL spectra (**a**) in the 0–7 ps and 8–36 ps temporal windows; (**b**) decay kinetic from the band edge (475–505 nm); (**c**) gated PL spectra in the 0–3 μs and 10–12.5 μs temporal windows; (**d**) decay kinetic from the first trap state (640–740 nm).

A group of sixteen commercially-available modern cadmium-based pigments, which are binary alloys of ZnS and CdS (Zn_1-x_Cd_x_S with 0 < x < 1), and of CdS and CdSe (CdS_x_Se_1-x_ with 0 < x < 1), were studied in the laboratory with the TRPL device opening new frontiers both for the characterization of the material itself and for the understanding of their degradation paths. Our attention focused on the photoluminescence emissions from the band edge (E_g_) and the first deep trap state (E_t1_) [19,42], respectively related to the crystal lattice and to defects and vacancies present in it. The time-resolved analysis permitted a deeper study of this class of pigments compared to the spectrally resolved results obtained in previous studies [6,42]. The gated spectra and the emission decay kinetics from one of the yellow shades are presented in Figure 6.

The band edge emission (E_g_), with a maximum peak between 476 nm and 630 nm for the various samples, has a lifetime on the order of picoseconds and can be used to discriminate between the two different pigment compositions, since there is at least a 10 ps difference in the τ, with emission from CdS pigments decaying more rapidly than that from CdSe. Secondly, the trap state emission (E_t1_), characterized by a λ_Peak_ between 660 nm and 800 nm, presents a μs decay profile, without a direct correlation with the pigments composition.

Other semiconductors found in paint are based on zinc calcogenides—the white pigments zinc oxide (ZnO), zinc sulphide (ZnS) and lithopone (ZnS + BaSO_4_), which were all produced on a massive scale during the 19th C and 20th C. Due to the intensity, purity, cost and covering power, zinc-based whites often outclassed the traditional pigment white lead. In-situ lifetime imaging was employed for the mapping of a white luminescent pigment present on Van Gogh's painting “Les bretonnes et le pardon de pont Aven” [43]. For these investigations, the gating window of the intensified camera was set to 100 ns, and the emission decay was recorded from 0 to 1,000 ns with a variable sampling step. The dynamics of the decay of the luminescent pigment, analysed with a multiexponential decay model assuming a maximum of three discrete components, highlighted a very long-lived emission at 520 nm with an effective lifetime close to 1,100 ns that we ascribe to traces of ZnS with the presence of Cu ions unintentionally introduced during the pigment manufacturing. Indeed, well into the 20th century metal impurities were often introduced into the crystal matrix of the pure substances depending on the method of synthesis and purification of raw materials.

### 3.3. Analysis of Works on Stone

The FLIM device has been applied to the analysis of marble sculptures, with most important studies including the investigation of two Michelangelo's masterpieces, the David and the Pietà Rondanini. In all cases, the time gated imaging camera was set in the nanosecond gating mode in order to filter out the long-living luminescence from marble. As was outlined in the introduction, minerals often emit a characteristic luminescence with lifetimes from microseconds to milliseconds, ascribable to impurities located inside the crystal lattice of the material, such as Mn^2+^ ions substituting for Ca^2+^ ions in calcite. This working principle allowed us the detection of the nanosecond emission from organic compounds which have been absorbed in the porous marble matrix. For example, analysis of different areas of Michelangelo's David revealed the presence of an intense nanosecond emission heterogeneously distributed on the sculpture surface. Areas with lifetimes close to 6 and 4.5 ns were detected and, with the aid of complementary Fourier Transform Infrared Spectroscopy (FTIR) of samples, allowed the mapping of beeswax (which has a longer lifetime), which permeates most of the statue surface, and of calcium oxalate deposits (shorter lifetime) in vertical patterns. The former organic contaminant was ascribed to a beeswax-based conservation treatment carried out in 1813 on the sculpture. Oxalate accumulation was likely due to the outdoor display of the David until 1873, when the statue was moved inside the museum (Galleria dell'Accademia, Florence, Italy) where it is today. The shorter mean lifetime from areas with the inorganic deposits could be due to traces of fluorescent compounds in the patina with a lifetime shorter than that of beeswax.

### 3.4. Applications to Polymer-Based Materials

Recently, both FLIM and TRPL have been extended to the monitoring of polymers and their degradation [44]. A range of polymers can be found in cultural heritage, and the changes in chemical properties of modern materials—whether those found in design objects or museum collections, or materials based on polymers which are used for conservation treatments (such as adhesives or consolidants)—are of particular concern. FLIM and TRPL are both useful tools for monitoring changes in luminescence which may develop or change with polymer degradation. For example, the photo-oxidation of acrylonitrile butadiene styrene (ABS) has been shown to lead to significant modifications of the fluorescence spectrum and the fluorescence lifetime. On the ps time scale, the lifetime of fluorescence related to styrene is reduced, which is ascribed to modifications of the molecular weight of ABS. In objects made of ABS, FLIM is useful for the identification of differences in formulation (and the presence, for example, of optical brighteners and other impurities) in objects made of different parts in ABS, as well as the build-up of longer-lived fluorophores (with lifetimes of *ca.* 5 ns) due to degradation.

## 4. Discussion

Time-resolved luminescence techniques applied to the analysis of cultural heritage provide a rapid means for differentiating materials, mapping distribution or, in some cases, detecting chemical changes. The real strength in FLIM is that it gives quantitative measurements with a portable and versatile instrument and the fact that new information, which complements steady-state fluorescence imaging, can be obtained regarding the nature of luminescent materials on a surface. The distribution of trace original materials (for example the fluorescent pigments in wall paintings by Masolino, or the phosphorescent white semi-conductor pigments present in an impressionist painting by Van Gogh) can be mapped rapidly and in conditions which do not require complete darkness, which is a distinct advantage for the in situ analysis of cultural heritage artworks. In other cases, added materials, like wax (in the case of the David) or organic binding media (for example shown in replicas of wall paintings) may be responsible for signal detected by FLIM. While ps-resolved analysis using a streak camera is confined to the laboratory, analysis of pigments and polymers demonstrates how fluorescence spectra shift, how fluorescence lifetime changes with degradation or how it may depend on pigment manufacture. Indeed, time-resolved fluorescence has proved to be an optimal sensor for specific materials (including semiconductors) found in cultural heritage.

Despite these advantages, the interpretation of changes in emission lifetime is complex, and it is made more so due to the heterogeneity intrinsic in artist materials and works of art. Indeed it is often useful to employ complementary elemental and molecular analyses for a better understanding of chemical differences associated with different time-resolved emissions. Some non-invasive instrumentation can be utilised for this purpose, and this has become more straightforward with the introduction of commercial non-destructive instrumentation, as has been shown in many projects (including, for example, the Cultural Heritage Advanced Research Infrastructures (CHARISMA) project [45]). Nonetheless, it is rarely possible to precisely ascribe modifications in fluorescence lifetime in complex materials without fundamental studies of reference materials which, albeit pure, are far removed from the complex surfaces encountered in works of art. Current research is focusing on fundamental studies of the interactions of organic and inorganic materials in model paintings using a range of instrumentation including TRPL.

Data analysis and processing are an important part of FLIM and TRPL—as is the careful selection of experimental conditions. In addition, the optoelectronic components which are part of the FLIM and TRPL device and the laser-excitation both limit the availability of the techniques for routine analysis. Technological advances in time-resolved detecting systems have recently been quite impressive, with the introduction of compact low-cost SPAD arrays for TCSPC measurements. Indeed, biological and medical applications could drive technology and the development of very compact and low cost time-resolved imaging devices.

## 4. Conclusions and Outlook

In this work applications of FLIM and TRPL to cultural heritage have been presented along with technical details regarding their implementation. The techniques can provide useful information about a range of inorganic and organic materials either in situ (FLIM) or in the laboratory (TRPL), as has been illustrated with applied case studies. While the data acquired may be difficult to interpret and ascribe, it can inform sampling or further analysis. Both techniques are ideally suited to monitoring as they are non-destructive and luminescence has been shown to change as a consequence of material degradation. Future applications of FLIM should focus on the study of a larger range of semiconductor materials, including historic samples, as well as on other long-lived luminescent materials (for example Egyptian blue) found in art. As the imaging device can be easily adapted to an optical microscope, analyses should be extended to the micro scale for the study of pigment particles. Finally, spectral extensions of the detection systems used for TRPL will allow the monitoring of oxidation of organic materials which emit in the UV (for example amino acids and small aromatic molecules present in binding media and plastics).

## Figures and Tables

**Table 1.** Emission lifetimes of typical luminescent materials found in cultural heritage.

**Material**	**Emitting Molecule or Process**	**Emission Lifetimes**
Protein and oil-based binders, varnishes, adhesives and glues	Organic molecules	ps to tens of ns
Organic natural and synthetic pigments	Organic molecules	ps to tens of ns
Semiconductor pigments	Band gap recombination Trap levels	ps to ns μs
Doped semiconductor pigments	Impurities	μs to hours
Minerals	Intrinsic or extrinsic defects in the crystal lattice	μs to ms
